# Supplementary material for: High-throughput multi-camera array microscope platform for automated 3D behavioral analysis of swimming zebrafish larvae
Source: bioRxiv. 2025 Oct 20:2025.07.07.661868. Originally published 2025 Jul 8. Preprint. [Version 2] doi: 10.1101/2025.07.07.661868 (PMC12265530; doi:10.1101/2025.07.07.661868)
Supplement: Supplement 1 [file NIHPP2025.07.07.661868v2-supplement-1.pdf]

bioRxiv preprint doi: https://doi.org/10.1101/2025.07.07.661868; this version posted October 20, 2025. The copyright holder for this preprint (which was not certified by peer review) is the author/funder, who has granted bioRxiv a license to display the preprint in perpetuity. It is made available under aCC-BY 4.0 International license.

S2 Additional figures

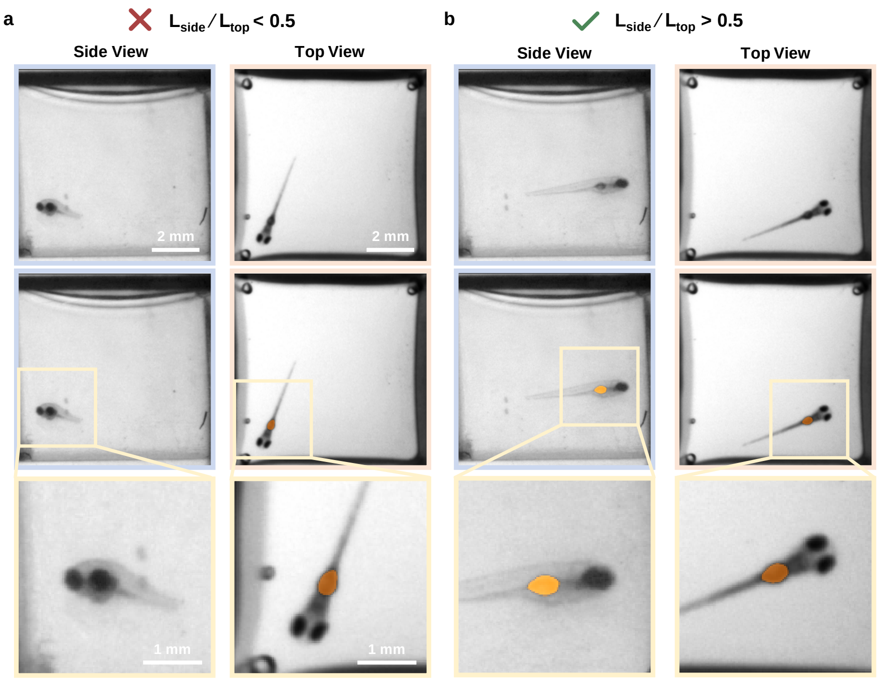

Fig. S1 Representative examples of our visibility criteria for segmenting the swimbladder. a, Visibility criteria is not met, the fishes projected length on the sideview is short relative to the true length, resulting in the swimbladder being behind the zebrafish's eyes. **b,** Visibility criteria is met, the long projected lengths in both views ensure that the swim bladder is clearly visible and segmentable.

bioRxiv preprint doi: https://doi.org/10.1101/2025.07.07.661868; this version posted October 20, 2025. The copyright holder for this preprint (which was not certified by peer review) is the author/funder, who has granted bioRxiv a license to display the preprint in perpetuity. It is made available under aCC-BY 4.0 International license.

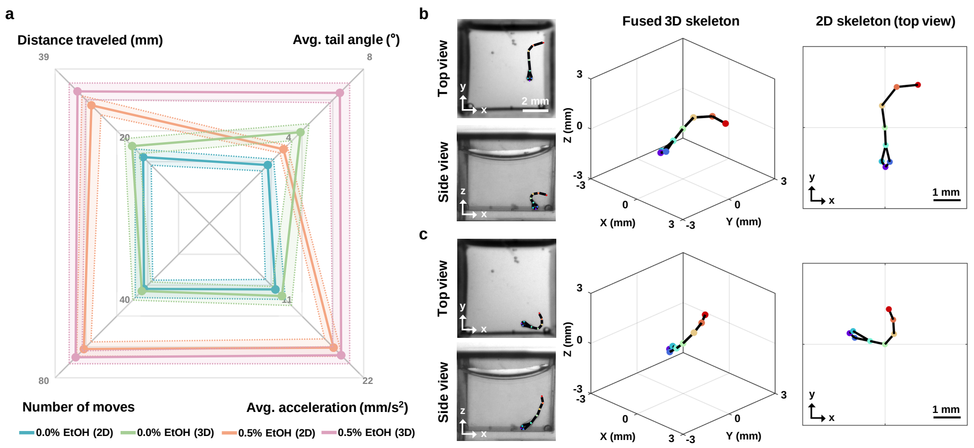

Fig. S2 Comparison of 2D and 3D kinematic analysis under ethanol exposure using multivariate visualization and skeletal reconstruction. a, Nightingale plot illustrating the average values and standard errors (SEM, shaded regions) of four key kinematic parameters for zebrafish larvae exposed to 0.0% or 0.5% ethanol (EtOH), measured using 2D and 3D tracking ($n = 8$ per group). **b-c,** Two examples of larvae exposed to 0.5% EtOH. In **b**, the top view suggests a classic J-turn, while the side view reveals that the movement is actually a C-turn with strong vertical curvature. In **c**, the side view reveals an upward arched tail bend not captured from above.
