# Supplementary material for: High-throughput multi-camera array microscope platform for automated 3D behavioral analysis of freely swimming zebrafish larvae
Source: bioRxiv. 2025 Jul 8:2025.07.07.661868. Preprint. [Version 1] doi: 10.1101/2025.07.07.661868 (PMC12265530; doi:10.1101/2025.07.07.661868)
Supplement: Supplementary file 1 [file NIHPP2025.07.07.661868v1-supplement-1.pdf]

bioRxiv preprint doi: https://doi.org/10.1101/2025.07.07.661868; this version posted July 8, 2025. The copyright holder for this preprint (which was not certified by peer review) is the author/funder, who has granted bioRxiv a license to display the preprint in perpetuity. It is made available under aCC-BY 4.0 International license.

Supplementary information

S1 Supplementary video

1. **System principles, 3D skeletal tracking, and swim bladder morphodynamics.**

bioRxiv preprint doi: https://doi.org/10.1101/2025.07.07.661868; this version posted July 8, 2025. The copyright holder for this preprint (which was not certified by peer review) is the author/funder, who has granted bioRxiv a license to display the preprint in perpetuity. It is made available under aCC-BY 4.0 International license.

S2 Additional figures

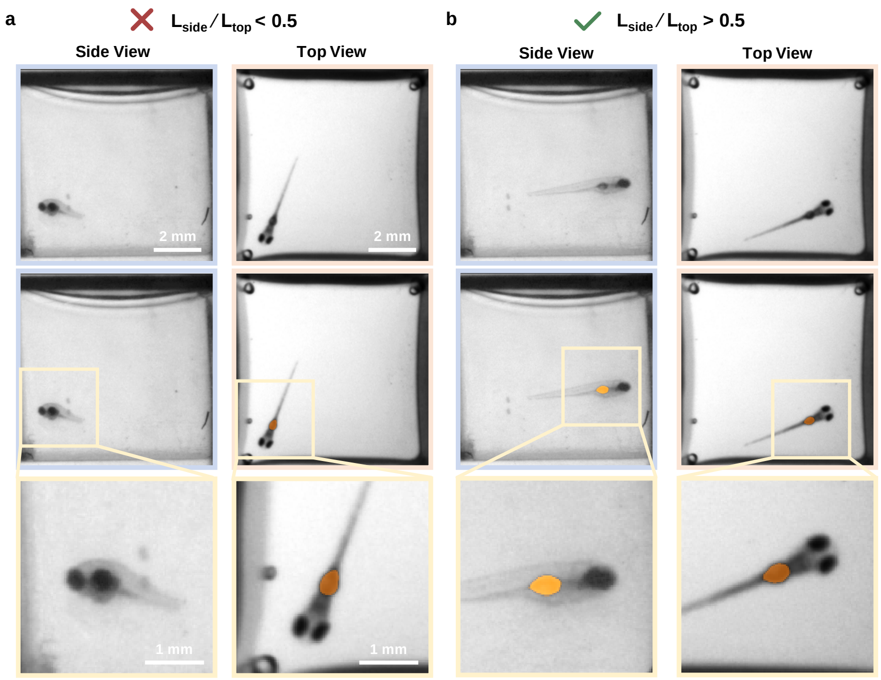

Fig. S1 Representative examples of our visibility criteria for segmenting the swimbladder. a, Visibility criteria is not met, the fishes projected length on the sideview is short relative to the true length, resulting in the swimbladder being behind the zebrafish's eyes. **b,** Visibility criteria is met, the long projected lengths in both views ensure that the swim bladder is clearly visible and segmentable.
